# Developing the Stroke Exercise Preference Inventory (SEPI)

**DOI:** 10.1371/journal.pone.0164120

**Published:** 2016-10-06

**Authors:** Nicholas S. Bonner, Paul D. O’Halloran, Julie Bernhardt, Toby B. Cumming

**Affiliations:** 1 School of Psychology and Public Health, La Trobe University, Bundoora, Victoria, Australia; 2 Florey Institute of Neuroscience and Mental Health, University of Melbourne, Melbourne, Victoria, Australia; University of Glasgow, UNITED KINGDOM

## Abstract

**Background:**

Physical inactivity is highly prevalent after stroke, increasing the risk of poor health outcomes including recurrent stroke. Tailoring of exercise programs to individual preferences can improve adherence, but no tools exist for this purpose in stroke.

**Methods:**

We identified potential questionnaire items for establishing exercise preferences via: (i) our preliminary Exercise Preference Questionnaire in stroke, (ii) similar tools used in other conditions, and (iii) expert panel consultations. The resulting 35-item questionnaire (SEPI-35) was administered to stroke survivors, along with measures of disability, depression, anxiety, fatigue and self-reported physical activity. Exploratory factor analysis was used to identify a factor structure in exercise preferences, providing a framework for item reduction. Associations between exercise preferences and personal characteristics were analysed using multivariable regression.

**Results:**

A group of 134 community-dwelling stroke survivors (mean age 64.0, SD 13.3) participated. Analysis of the SEPI-35 identified 7 exercise preference factors (*Supervision-support*, *Confidence-challenge*, *Health-wellbeing*, *Exercise context*, *Home-alone*, *Similar others*, *Music-TV*). Item reduction processes yielded a 13-item version (SEPI-13); in analysis of this version, the original factor structure was maintained. Lower scores on *Confidence-challenge* were significantly associated with disability (p = 0.002), depression (p = 0.001) and fatigue (p = 0.001). Self-reported barriers to exercise were particularly prevalent in those experiencing fatigue and anxiety.

**Conclusions:**

The SEPI-13 is a brief instrument that allows assessment of exercise preferences and barriers in the stroke population. This new tool can be employed by health professionals to inform the development of individually tailored exercise interventions.

## Introduction

Physical activity is essential to post-stroke recovery, with evidence that exercise training improves functional capacity, increases quality of life and reduces the risk of subsequent cardiovascular events.[1] The benefits of physical activity after stroke are many and varied, ranging from increased cardiorespiratory fitness[2] to a reduction in depressive symptoms.[3] Yet many stroke survivors in the community are physically inactive, with step counts, energy expenditure and self-reported physical activity well below recommended levels.[4] A key problem is that stroke survivors who initiate exercise programs fail to maintain engagement in the longer term.[5] Throughout this paper, the terms ‘physical activity’ and ‘exercise’ will be used interchangeably to denote any bodily movement produced by skeletal muscles that substantially increases energy expenditure over resting levels.[6]

Evidence indicates that individual tailoring is a feature of effective interventions for increasing physical activity, both in general[7] and stroke[8] populations. Current exercise tailoring practices in stroke are typically limited to consideration of physical capability, and inclusion of personalised goal setting and counselling.[8] A more comprehensive conception of individual tailoring includes aspects such as preferred environment, level of supervision, social support and type of exercise activity.[1] When exercise conditions are more congruent with personal preferences, affective responses are more positive.[9] This is important as positive affect during exercise has been linked to greater intention to exercise[10] and future exercise behaviour.[11] Identifying and incorporating individual exercise preferences may be particularly important in stroke given the heterogeneous nature of disability, the high number of exercise barriers[12] and the high variability in preferred exercise conditions.[13] In other medical populations (e.g., cancer survivors,[14] cardiac patients[15]), exercise preference scales have been developed and used to overcome barriers to participation.

At present, no instruments exist for assessing exercise preferences in stroke survivors. Our primary aim was to develop a new questionnaire, the Stroke Exercise Preference Inventory (SEPI), to evaluate exercise preferences and barriers after stroke. A secondary aim was to determine the relationship between key personal characteristics (disability, fatigue, depression, anxiety) and self-reported exercise preferences and barriers, in order to evaluate whether these characteristics could account for individual differences on the SEPI.

## Methods

### Study design

The Stroke Exercise Preference Inventory (SEPI) was developed in two stages: content development and content refinement. Stage 1 involved identifying a wide range of questionnaire items that covered meaningful aspects of exercise preferences after stroke. Once these items were finalised, Stage 2 involved administering them to a sample of stroke survivors and analysing the data to refine the questionnaire to a core set of items.

### Stage 1 –Content development

To begin the development process, we built a list of potentially relevant questionnaire items. These items were drawn from multiple sources, including our preliminary Exercise Preference Questionnaire,[13] a review on exercise barriers and facilitators in stroke,[12] and exercise preference questionnaires developed for other populations.[14,15] Aiming to be inclusive to cover the broadest possible range of exercise preferences, we identified 39 items.

To further develop and ratify items that were relevant, easily comprehended and unambiguous, we convened an expert panel.[16] Members of the panel were invited on the basis that they had either: (a) experience in working with stroke survivors in an exercise context, or (b) specialist academic knowledge of stroke or exercise. The panel consisted of 3 Melbourne-based senior clinician-researchers (a neurologist with more than 10 years’ experience in clinical stroke care, a physiotherapist and an exercise physiologist, both with 20 years’ experience in prescribing exercise to stroke rehabilitation inpatients) and 2 international senior clinician-researchers (a physiotherapist with more than 10 years’ experience in exercise testing after stroke and a geriatrician with more than 20 years’ experience in clinical stroke care who is a research leader in post-stroke exercise guidelines). The Melbourne-based experts participated in a 2-hour panel discussion with the research team. Part 1 of the discussion was a brainstorming session where panel members were asked to focus on what stroke survivors like and dislike in exercise programs, and what common barriers and facilitators exist. In part 2 of the discussion, panel members were given a copy of the 39-item list and asked to independently rate the importance of each item to the understanding of exercise preferences after stroke (from 1 –‘not important’ to 4 –‘essential’). The international experts contributed written suggestions and feedback via email using the same 2-part format. Following completion of the expert panel discussion and email correspondence, we collated all the exercise preference items and all the barrier items that had been identified. The investigator team then met to select a final list of items, driven by the principles of remaining inclusive and keeping all items deemed to be relevant to stroke survivors, but also by eliminating any redundancy in the item pool. Final agreement across at least 3 of the 4 members of the investigator team was required before any item was removed from the pool. Some changes to the wording of included items were made at this point. The content development process resulted in the SEPI-35, which included 35 exercise preference items and 9 exercise barrier items.

### Stage 2 –Content refinement

#### Participants

Community-dwelling stroke survivors were included if they were aged ≥18 years and had sufficient English language comprehension. There was no limit placed on time since stroke. Participants with transient ischemic attack (TIA) were excluded. To maximise generalisability, participants were not excluded on the basis of disability severity or co-morbid health conditions. Participants were recruited via multiple settings, including a hospital stroke outpatient clinic (Austin Health), a rehabilitation hospital (Royal Talbot Rehabilitation Centre), community-based stroke support groups from around Australia, and through the National Stroke Foundation of Australia website. The study was approved by the Human Research Ethics Committee of Austin Health, and all participants provided written informed consent.

#### Procedure

Participants completed the SEPI-35 and other assessments in a single session, either face-to-face at the point of recruitment or remotely via mailed out questionnaire packs. For the remote completers, phone contact was always made to ensure data integrity and completeness. No data were recorded on participant response rates, as numerous questionnaire packs were supplied to interstate support groups, making it difficult to accurately track the number of potential responders.

#### Outcome measures

A 1-page questionnaire featuring demographic (age, sex, marital status, living arrangements, postcode, education) and stroke-related (stroke date, type, side of symptoms) questions was administered. The primary outcome was the SEPI-35, which consisted of 35 items on preferences for exercise and 9 items on barriers to exercise participation. Examples of preference items included: ‘I like to exercise outdoors’ and ‘I like a trained instructor to supervise my exercises’. Participants were asked to indicate their level of agreement with each item by choosing a number between 0% (‘Don’t agree at all’) and 100% (‘Totally agree’). The modified Rankin Scale (mRS) is a 7-point scale assessing disability, ranging from 0 (no symptoms) to 6 (death). It is widely used in stroke research, with good predictive validity and inter-rater reliability.[17] To score the mRS, we used a 3–5 minute structured interview (either face-to-face or by telephone), which has been shown to enhance measurement reliability.[18] The Patient Health Questionnaire (PHQ-9) is a 9-item depression screening tool that is scored from 0–27; it has good validity against a clinical diagnosis of depression in stroke.[19] The Generalised Anxiety Disorder screening tool (GAD-7) contains 7 items and is scored from 0–21; it is valid for assessing anxiety.[20] The Fatigue Assessment Scale (FAS) is a 10-item measure of fatigue that is scored from 0–50; it has been recommended for use in stroke patients.[21] On all 3 of these scales, higher scores indicate greater burden of symptoms (depression, anxiety or fatigue). The International Physical Activity Questionnaire (IPAQ) short-form is a 7-item measure of self-reported physical activity that has reasonable validity and test-retest reliability.[22]

### Statistical analysis

There is no consensus on a minimum sample size for valid exploratory factor analysis, although there is agreement that the larger the N and N:item ratio the better.[23] Several authors have suggested a minimum participant to item ratio of 5:1, although others suggest that >50 but <100 total participants is adequate.[24] We set a target sample size of 140, reflecting a 4:1 participant:item ratio on the SEPI-35 exercise preference items. A sample size of 105 (3:1 ratio) was deemed acceptable as a minimum standard.

Exploratory Factor Analysis using Principal Components Analysis was employed to identify the factor structure of the SEPI-35, with eigenvalues >1 extracted. Bartlett’s test of sphericity and Kaiser-Meyer-Olkin sampling adequacy were used to assess the reliability of the factor structure. In order to identify the most logical data structure, several factor rotations were examined, including varimax, direct oblimin, quartimax, equamax and promax.

Item removal from the SEPI-35 was based on 4 guiding principles, informed by statistical evidence. The first principle was strength of factor loading. Items were removed if they did not load above 0.50 on any factor. Items with higher factor loadings were prioritised for selection in the reduced item pool. The second principle was strength of internal reliability. Items were favoured if they improved the internal reliability of a factor, as assessed using the Cronbach’s alpha and ‘Cronbach’s alpha if item deleted’ statistics for each item in a factor. The third principle was conceptual similarity. Within each factor, items deemed too conceptually similar were considered for removal. These judgements were weighed against the second principle, as conceptually similar items often had strong scale reliability properties. The fourth principle was discrimination strength. As per Item Response Theory, items that were better at discriminating between high and low scorers on a given factor were favoured for selection.[25] To quantify this, total factor scores were split into quartiles and, for each item within that factor, mean item scores were compared between individuals in the top and bottom quartiles. Items with greater mean difference were preferred, as they were considered a better reflection of individual differences in exercise preferences on that factor.

Once the reduced pool of SEPI items had been finalised (the SEPI-13, see S1 Appendix), we ran a Principal Components Analysis on these items to establish whether the SEPI-35 factor structure was maintained. Statistical conditions for this second Principal Components Analysis were kept consistent with the previous SEPI-35 analysis, with the number of factors fixed, method of rotation the same, and loadings <0.50 suppressed.

To evaluate variability in factor scores, means and standard deviations were calculated for all 7 SEPI-13 factors. To examine associations between personal characteristics and exercise preferences, we calculated standardised mean factor scores (z-scores, weighted by factor loadings). First, we established whether age and sex were associated with scores on each factor using multivariable linear regressions. Additional regressions, adjusting for age and sex, were used to determine the association between each exercise preference factor score (as the dependent variable) and disability, depression, anxiety and fatigue (as the independent variable). For the purpose of representing the data in spider plots, participants were dichotomised on each of these variables: disabled (mRS≥3) or not, depressed (PHQ-9≥10) or not, anxious (GAD-7≥5) or not, fatigued (FAS≥25) or not.

Data from the exercise barrier items, not our main focus here, were summarised using descriptive statistics. The relationship between personal characteristics (disability, depression, anxiety, fatigue) and the exercise barrier items was examined by computing Spearman correlation coefficients.

## Results

Data on demographics, stroke details and other outcome measures for the 134 participants are presented in Table 1. In terms of recruitment setting, 98 (73%) were from stroke support groups, 14 (10%) from the Royal Talbot Rehabilitation Centre, 13 (10%) from a National Stroke Foundation internet call-out, 7 (5%) from the Austin Repatriation Hospital, and 2 (1%) from word of mouth.

**Table 1:** Demographic and stroke characteristics of the participants.

	Stroke (n = 134)
Age: mean (SD)	63.8 (13.3)
Sex, male	75 (56%)
Years since stroke: mean (SD), *median (IQR)*	8.4 (9.0), *6*.*4 (2*.*0–11*.*8)*
Stroke type–infarct	43 (32%)
–haemorrhage	44 (33%)
–both	2 (1%)
–don’t know	37 (28%)
–did not respond	8 (6%)
Side affected–left	66 (49%)
–right	46 (34%)
–both	4 (3%)
–neither	10 (7%)
–did not respond	8 (6%)
Living–home with others	101 (75%)
–home alone	26 (19%)
–other	4 (3%)
–did not respond	3 (2%)
Marital status–married	72 (54%)
–divorced	26 (19%)
–single	17 (13%)
–other	17 (13%)
–did not respond	2 (1%)
mRS– 0	2 (1%)
– 1	21 (16%)
– 2	44 (33%)
– 3	51 (38%)
– 4	15 (11%)
–missing	1 (1%)
Depression: mean (SD), *median (IQR)*	6.6 (5.7), *5 (2–11)*
–PHQ-9 ≥10	39 (31%)
Anxiety: mean (SD), *median (IQR)*	5.1 (5.2), *3 (1–8)*
–GAD-7 ≥5	57 (43%)
Fatigue: mean (SD), *median (IQR)*	25.0 (8.7), *24 (18–31)*
–FAS ≥25	64 (48%)
Exercise beyond daily activities–yes	90 (67%)
–no	42 (31%)
–did not respond	2 (1%)

### Factor analyses

Eight cases were excluded due to missing data. Bartlett’s test of sphericity was significant (χ^2^ = 2499.5, df = 595, p<0.01) and Kaiser-Meyer-Olkin sampling adequacy (0.85) was above the necessary level (0.60), indicating a strong likelihood of producing a distinct and reliable factor structure. Evaluated against our *a priori* estimation of factor groupings, the varimax rotation method produced the most logical factor structure. Factor analysis yielded 7 factors with eigenvalues >1, explaining 64% of total variance (see Table 2). Factor 1 accounted for 17%, factor 2 for 13%, and factor 3 for 8% of total variance. Three items failed to load on any factor at >0.50. These were ‘I like to do the same activities each time I exercise’, ‘I like to exercise at a gym or fitness centre’, and ‘I want to get back to doing the exercise I did before the stroke’. These items were removed from subsequent analysis. Factor labels were applied to each factor, based upon interpretation of item groupings (Table 2).

**Table 2:** Item loadings from exploratory factor analysis of the SEPI-35 (varimax rotation with Kaiser normalisation), n = 126, coefficients <0.50 are suppressed.

Factor label	Item	1	2	3	4	5	6	7
**Supervision-support**	I like a trained instructor to supervise my exercise	0.863						
	When I exercise I like someone being on hand to help if needed	0.809						
	I like to get feedback on how I'm going with my exercise	0.807						
	I like to have someone there encouraging me during exercise	0.781						
	I like someone showing me what to do when I exercise	0.751						
	I like someone else to organise my exercise sessions	0.710						
	I like exercise sessions to be planned in advance	0.638						
	I like to have written instructions for my exercise	0.524						
**Confidence-challenge**	I am confident I can get up and start exercising without delaying or making excuses		0.762					
	I like to be challenged by exercises		0.724					
	I am confident I can do the exercise I want to do		0.719					
	I am confident I can stay involved in a regular exercise program		0.691					
	I like to work hard when I exercise		0.677					
	I like to exercise		0.609					
	I like to have exercise goals		0.594					
**Health-wellbeing**	I like to exercise for health reasons			0.676				
	It is important for me to do exercise that makes me feel good			0.662				
	I like to make exercise part of my daily activities			0.575				
	I think exercise will help prevent another stroke			0.564				
	I think it is important for me to exercise			0.525				
**Exercise context**	I like to exercise with family or friends				0.670			
	I like to exercise for enjoyment or relaxation				0.618			
	I like to exercise in the morning				0.558			
	I like to exercise outdoors				0.535			
**Home-alone**	I like to exercise at home					0.804		
	I like to exercise alone					0.669		
	I like exercises to be focused on my stroke related deficits					0.519		
**Similar others**	I like to exercise in a community group						0.732	
	I like to exercise with other people of a similar age						0.660	
	I like to exercise with other people who have had a stroke						0.644	
	I like to exercise with other people of the same gender						0.501	
**Music-TV**	I like to listen to music or watch TV during exercise							0.813

Item reduction processes resulted in a set of 13 items, 2 each for the first 6 factors, and 1 for the 7^th^ factor. Analysis of scores on these 13-items (the SEPI-13)–with varimax rotation and number of factors fixed at 7 –showed that the original factor structure from the SEPI-35 was replicated (see Table 3). The 7 factors explained 83% of total variance on the SEPI-13, with factor 1 accounting for 30%, factor 2 for 18%, and factor 3 for 9%. Bartlett’s test of sphericity was significant (χ^2^ = 580.1, df = 78, p<0.01) and Kaiser-Meyer-Olkin sampling adequacy was strong (0.75). Unweighted factor scores (means and standard deviations) are shown in Table 3.

**Table 3:** Item loadings from exploratory factor analysis of the SEPI-13 (varimax rotation with Kaiser normalisation, fixed at 7 factors), n = 126, coefficients <0.50 are suppressed. Unweighted factor scores (% agreement) are shown as mean and standard deviation.

Factor label	Item	1	2	3	4	5	6	7	Mean (SD)
**Supervision-support**	I like a trained instructor to supervise my exercise	0.901							56 (37)
	I like to get feedback on how I'm going with my exercise	0.844							
**Confidence-challenge**	I am confident I can stay involved in a regular exercise program		0.863						58 (33)
	I like to be challenged by exercises		0.789						
**Health-wellbeing**	I like to exercise for health reasons			0.817					80 (27)
	It is important for me to do exercise that makes me feel good			0.800					
**Exercise context**	I like to exercise with family or friends				0.864				46 (31)
	I like to exercise outdoors				0.750				
**Home-alone**	I like to exercise at home					0.941			49 (32)
	I like to exercise alone					0.748			
**Similar others**	I like to exercise with other people of a similar age						0.821		42 (33)
	I like to exercise with other people who have had a stroke						0.749		
**Music-TV**	I like to listen to music or watch TV during exercise							0.950	44 (40)

### SEPI-13 regression analyses

Older age was associated with higher factor z-scores on *Similar others* (B = 0.014, se = 0.007; *p* = 0.045), *Home-alone* (B = 0.018, se = 0.007; *p* = 0.006) and lower scores on *Music-TV* (B = -0.018, se = 0.007; *p* = 0.008). Female sex was related to higher z-scores on *Home-alone* (B = 0.369, se = 0.174; *p* = 0.037).

The following multivariable regressions were adjusted for age and sex. People with more disability had a significantly higher preference for *Supervision-support* (B = 0.202, se = 0.098; *p* = 0.042) and scored lower on *Confidence-challenge* (B = -0.293, se = 0.093; *p* = 0.002). People with more depressive symptoms had a significantly higher preference for *Music-TV* (B = 0.035, se = 0.016; *p* = 0.030) and scored lower on *Confidence-challenge* (B = -0.056, se = 0.016; *p* = 0.001). People with more anxiety symptoms had a (non-significantly) stronger preference to exercise with *Similar others* (B = 0.033, se = 0.017; *p* = 0.055). People with more fatigue had significantly lower scores on the *Confidence-challenge* factor (B = -0.048, se = 0.009; *p*<0.001).

**Fig 1:**
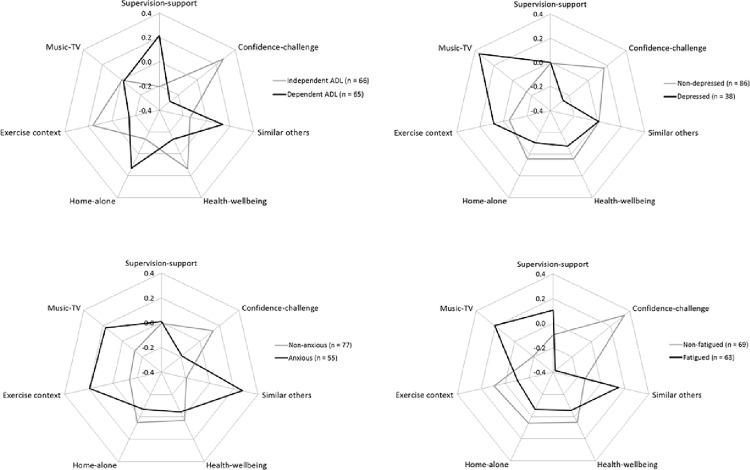
**Spider plots of weighted factor scores on each of the 7 SEPI-13 factors according to status on [A] disability, [B] depression, [C] anxiety and [D] fatigue.**

The associations between personal characteristics (disabled or not, depressed or not, anxious or not, fatigued or not) and each of the exercise preference factors (factor z-scores weighted according to factor analysis loadings) are visually represented in Fig 1.

### Exercise barriers

Means and standard deviations for each barrier item are presented in Table 4, in descending order, along with proportions of the sample reporting 0% agreement, 0–49% agreement, and ≥50% agreement with each item. Correlations between personal characteristics (disability, depression, anxiety, fatigue) and the barrier items are outlined in Table 5.

**Table 4:** Means, standard deviations and percentages of those reaching varying thresholds of agreement for the 9 exercise barrier items.

Barrier item	Mean	SD	%0	%1–49	%50+
Even though I want to exercise I find it hard to get started	35.7	36.2	34	25	41
I worry that I'll fall if I exercise	30.9	39.4	43	24	33
I find it hard to get to places where I want to exercise	28.6	37.4	50	18	32
I feel too tired to exercise	28.0	34.7	44	25	31
I don't have enough information about the exercise I should be doing	27.6	37.6	51	18	31
The exercise I want to do is too expensive	21.8	32.7	58	14	27
I avoid exercise because it causes me pain	19.7	33.3	61	18	21
I worry that exercise might cause another stroke	13.2	26.3	69	16	15
I avoid exercise because its unsafe	12.5	25.9	71	15	14

**Table 5:** Spearman correlation coefficients and statistical significance for the association between each barrier item and disability, depression, anxiety and fatigue.

Barrier item	Disability	Depression	Anxiety	Fatigue
I worry that exercise might cause another stroke	.034	.217^*^	.313^**^	.157
The exercise I want to do is too expensive	.094	.135	.209^*^	.128
I avoid exercise because it causes me pain	.232^**^	.288^**^	.344^**^	.313^**^
I don't have enough information about the exercise I should be doing	-.024	.150	.205^*^	.195^*^
I worry that I'll fall if I exercise	.324^**^	.433^**^	.440^**^	.408^**^
I find it hard to get to places where I want to exercise	.235^**^	.191^*^	.254^**^	.227^**^
I avoid exercise because its unsafe	.182^*^	.242^**^	.242^**^	.271^**^
I feel too tired to exercise	.360^**^	.402^**^	.407^**^	.551^**^
Even though I want to exercise I find it hard to get started	.313^**^	.388^**^	.398^**^	.565^**^

*p<0.05

**p<0.01

## Discussion

We developed a comprehensive 35-item exercise preference inventory (SEPI-35) through expert consultation, and identified 7 discernible exercise preference factors relevant to the stroke population. The factor structure of the SEPI-35 was maintained in a refined 13-item version (SEPI-13), providing a parsimonious measure for assessing key exercise preferences in the stroke population (see S1 Appendix). In addition to the 13 preference items, the questionnaire features an optional 9-item module on barriers to exercise participation after stroke. Our results indicate multiple dimensions in exercise preferences, and reveal high individual variability in preferred exercise conditions, highlighting the need for tailored post-stroke exercise programs. As the first stroke-specific tool for assessing exercise preferences, the SEPI can be used by both researchers and clinicians.

The 7 exercise preference factors were: 1) *Supervision-support*, 2) *Confidence-challenge*, 3) *Health-wellbeing*, 4) *Similar others*, 5) *Exercise context*, 6) *Home-alone*, and 7) *Music-TV*. For some, receiving professional supervision and support can engender feelings of control during exercise and reduce safety-related fears.[26] For others, however, supervision and support may only serve to undermine a desire for independence and autonomy.[12] In our cohort, participants with more disability preferred higher levels of supervision and support. The *Confidence-challenge* factor assesses self-confidence in initiating and maintaining exercise, and the desire to be challenged. These two concepts are both closely related to exercise self-efficacy.[27] Making exercise difficulty and intensity congruent with personal confidence may promote feelings of control and mastery, factors which promote intrinsic motivation to exercise. Lower scores on *Confidence-challenge* were linked to disability, depression and fatigue. This suggests that optimal challenge in an exercise program should be tailored on the basis of both physical and psychological considerations. The *Similar others* factor evaluates the desire to exercise with people who share important characteristics, such as medical condition or age. Such contact may be valued as a means of obtaining social support, exchanging information, or minimising the risk of negative evaluation.[12] We identified a trend for participants with higher levels of anxiety to express a greater preference towards exercising with similar others. The *Home-alone* factor has clear implications for evaluating appropriateness of a home-based exercise program. Older age was found to be related to a preference for home-based exercise, which is consistent with previous findings.[28] The *Music-TV* factor was based on a single item. Participants with more depressive symptoms expressed a greater preference for music or TV during exercise. This may function as a source of pleasurable entertainment to boost positive affect and reduce negative affect during exercise,[29] or be valued as a form of dissociation to minimise pain or discomfort.[30]

There was marked individual variability across all exercise preference factors, with the exception of *Health-wellbeing* (see Table 3). For the *Health-wellbeing* factor, participants typically had a high level of agreement and relatively little variability (mean = 80%, SD = 27%). For the other 6 factors, level of agreement fell in the middle of the 0–100% scale (all means between 40% and 60%), with substantial variability (all SDs between 30% and 40%). With such large individual differences in exercise preferences, it is unsurprising that one-size-fits-all exercise programs suffer from problems with uptake and adherence.

Regarding exercise barriers, finding it hard to get started was the most commonly reported issue. This item had a particularly strong correlation with fatigue, which we know is prevalent after stroke.[31] Few participants reported avoiding exercise because of pain, safety fears or worry that it may cause another stroke. Exercise barriers were found to be more common in people experiencing psychological problems, with anxiety showing significant correlations with all 9 barrier items. The pattern of results suggests that knowledge of a person’s mental health may be important for explaining the existence of exercise barriers. Psychological conditions may underpin a pervasive range of fears, worries, and concerns about exercise behaviour.

The present study has limitations. We cannot exclude the possibility of error, particularly related to overfitting of the data; this possibility would be minimised in a larger sample. A larger sample size would also have allowed more robust forms of validation of the factor structure (e.g., an internal replicability analysis). Our data were collected both face-to-face and remotely, and it is possible that responses differed by mode of collection. Despite sample characteristics indicating good population representation with respect to demographic, stroke, and physical and mental health characteristics, we did have disproportionately high recruitment from community-based stroke support groups (73%) and this may have contributed to selection bias. There was also a relatively high percentage of people (67%) reporting exercise beyond daily activities; this sample may have been more active than a typical chronic stroke group. We need to confirm the stability of the tool when used in patients much earlier in the stroke recovery process, when the development of exercise habits may play an important role in recovery and prevention of further stroke.

### Summary

The SEPI-13 is a brief new instrument which can be used to assess the exercise preferences of stroke survivors. We have developed it for use in physical rehabilitation and exercise counselling settings as a tool to promote dialogue about exercise and inform program design. The SEPI-13, which can be administered without prior training, is likely to be most relevant to exercise physiologists, physiotherapists and exercise counsellors. It may assist in identifying meaningful facets of exercise experience that could otherwise go unmentioned. Regarding implementation, we are currently evaluating the feasibility and usefulness of the SEPI-13 in an inpatient rehabilitation setting. The SEPI-13 also has research applications, and could be used to improve our understanding of the relationship between individual exercise preferences and adherence to exercise programs after stroke.

## Supporting Information

S1 Appendix The Stroke Exercise Preference Inventory (SEPI). (DOC)

S1 Data Source data file. (SAV)
