# Supplementary material for: Developing the Stroke Exercise Preference Inventory (SEPI)
Source: PLoS One. 2016 Oct 6;11(10):e0164120. doi: 10.1371/journal.pone.0164120 (PMC5053595; doi:10.1371/journal.pone.0164120)
Supplement: S1 Appendix — (DOC) [file pone.0164120.s001.doc]

SEPI

Stroke Exercise Preference Inventory

- This questionnaire is about what kinds of exercise you like and don’t like.
- There is also a section on things that may stop you from exercising.
- Your answers will help us understand more about the best kinds of exercise for you.

__________________________________________________________________________

1. **Exercise Preferences**

Please indicate how much you agree with each of the following statements:

**Don’t agree at all Totally agree**

**0% 100%**

1. I like a trained instructor to supervise my exercise %
2. I am confident I can stay involved in a regular exercise program %
3. I like to exercise with other people who have had a stroke %
4. It is important for me to do exercise that makes me feel good %
5. I like to exercise at home %
6. I like to exercise outdoors %
7. I like to listen to music or watch TV during exercise %
8. I like to get feedback on how I’m going with my exercise %
9. I like to be challenged by exercises %
10. I like to exercise with other people of similar age %
11. I like to exercise for health reasons %
12. I like to exercise alone %
13. I like to exercise with family or friends %

_________________________________________________________________________

_________________________________________________________________________

1. **Potential Barriers**

Please indicate how much you agree with each of the following statements:

**Don’t agree at all Totally agree**

**0% 100%**

1. I worry that exercise might cause another stroke %
2. The exercise I want to do is too expensive %
3. I avoid exercise because it causes me pain %
4. I don’t have enough information about the exercise I should be doing %
5. I worry that I will fall if I exercise %
6. I find it hard to get to places where I want to exercise %
7. I avoid exercise because I think it’s unsafe %
8. I feel too tired to exercise %
9. Even though I want to exercise, I find it hard to get started %

_________________________________________________________________________

**Exercise Preferences scoring template**

Below is the scoring template for the 7 exercise preference factors. Use scores from part A to calculate average factor scores on factors 1-6 (factor 7 is a single item score). Means and standard deviations for each factor are provided as a reference (taken from a sample of 134 chronic stroke survivors).

1. **Supervision-support** Q1 ____ + Q8 ____ = **____ /** 2 **= ____** [mean=56, SD=37]

Higher score = preference for a trained instructor, getting feedback, having someone on hand to help if needed.

1. **Confidence-challenge** Q2 ____ + Q9 ____ = **____ /** 2 **= ____** [mean=58, SD=33]

Higher score = preference for being challenged, working hard, greater confidence to start and stay involved in exercise.

1. **Health-wellbeing** Q3 ____ + Q10 ____ = **____ /** 2 **= ____** [mean=80, SD=27]

Higher score = preference for exercise for health, that makes me feel good, as part of my daily activities.

1. **Exercise context** Q4 ____ + Q11 ____ = **____ /** 2 **= ____** [mean=46, SD=31]

Higher score = preference for exercise with family or friends, outdoors, for relaxation or enjoyment.

1. **Home-alone** Q5 ____ + Q12 ____ = **____ /** 2 **= ____** [mean=49, SD=32]

Higher score = preference for exercise at home, exercising alone.

1. **Similar others** Q6 ____ + Q13 ____ = **____ /** 2 **= ____** [mean=42, SD=33]

Higher score = preference for exercise with people of similar age, other stroke survivors, in a community group.

1. **Music-TV** Q7 ____ [mean=44, SD=40]

Higher score = preference for listening to music or watching TV during exercise.
